# Rethinking PDAC: from immunological silence to therapeutic opportunity

**DOI:** 10.3389/fimmu.2026.1909191

**Published:** 2026-08-11

**Authors:** Suresh K. Kali, Remya P. G. Ramesh, Aftab Alam, Maria J. Fernandez-Cabezudo, Aydah M. Al-Awadhi, Uday Kishore, Basel K. Al-Ramadi

**Affiliations:** 1 Department of Medical Microbiology and Immunology, College of Medicine and Health Sciences, United Arab Emirates University, Al Ain, United Arab Emirates; 2 Department of Veterinary Medicine, United Arab Emirates University, Al Ain, United Arab Emirates; 3 Department of Biochemistry and Molecular Biology, College of Medicine and Health Sciences, United Arab Emirates University, Al Ain, United Arab Emirates; 4 Zayed Center for Health Sciences, United Arab Emirates University, Al Ain, United Arab Emirates; 5 Division of Hematology and Oncology, Sheikh Shakhbout Medical City, Abu Dhabi, United Arab Emirates; 6 Department of Internal Medicine, College of Medicine and Health Sciences, United Arab Emirates University, Al Ain, United Arab Emirates; 7 ASPIRE Precision Medicine Research Institute Abu Dhabi, United Arab Emirates University, Al Ain, United Arab Emirates

**Keywords:** biomarker stratification, immune checkpoint blockade, neoantigen vaccine, pancreatic ductal adenocarcinoma, personalized immunotherapy, stromal modulation, tumor microenvironment

## Abstract

Pancreatic ductal adenocarcinoma (PDAC) ranks among the deadliest malignancies, with only 13% of patients surviving five years after diagnosis. This poor prognosis stems from late detection, treatment resistance, spatial and phenotypic heterogeneity, and a highly immunosuppressive tumor microenvironment. Unlike immunogenic cancers, PDAC is an immunologically ‘cold’ tumor, marked by minimal immune cell infiltration, defective antigen-presentation machinery, and stromal barriers. While immune checkpoint blockade (ICB) has shown success against various cancers, it has proven largely ineffective when used alone in PDAC. However, emerging research has identified promising new approaches, including neoantigen-targeted vaccines, stromal modulation, and combination therapies such as ICB with chemotherapy, cytokine blockade, and kinase inhibitors. The development of personalized immunotherapy approaches is being refined by predictive biomarkers that assess factors such as T cell infiltration levels, antigen-presenting capacity, and the spatial immune cell landscape. Here, we review the immune landscape of PDAC, current and emerging immunotherapeutic strategies, and highlight the critical role of biomarkers and immune profiling in appropriately categorizing patients. Collectively, these insights provide a foundation for designing rational combination therapies to transform PDAC into a cancer that is more immunologically responsive and amenable to therapy.

## 1 Introduction

Pancreatic ductal adenocarcinoma (PDAC) is one of the most aggressive cancers worldwide, with a 5-year survival of only 13% after diagnosis. According to the GLOBOCAN survey, pancreatic cancer accounted for approximately 460, 000 deaths and 510, 000 cases in 2022 (1). Most patients are diagnosed at an advanced stage and show heightened resistance to conventional therapies such as chemotherapy and radiotherapy (2–4). Delayed diagnosis, early metastasis, and highly immunosuppressive tumor microenvironment (TME) lead to poor prognosis in PDAC patients (5, 6). Unlike immunologically ‘hot’ tumors such as melanoma and non-small cell lung cancer, PDAC is predominantly classified as an immunologically ‘cold’ tumor due to its low mutational burden, aberrant antigen presentation, and restricted immune infiltration (7–9). Recent multi-omics and single-cell approaches have revealed that PDAC tumors display significant heterogeneity in their immune landscape. While a subset of tumors exhibits a myeloid-enriched phenotype with poor clinical outcomes, another subset exhibits an adaptive immune cell-enriched phenotype with better clinical outcomes (10–12). These findings highlight the importance of immune cell composition and infiltration in determining clinical outcomes in PDAC patients. Furthermore, PDAC is characterized by a dense fibrotic and desmoplastic stroma that comprises cancer-associated fibroblasts (CAFs), pancreatic stellate cells (PSCs), extracellular matrix proteins, and immune-modulating cytokines (13, 14). These stromal cells physically block effector CD8^+^ T cells from interacting with tumor cells and promote immune evasion by secreting immunosuppressive chemokines and cytokines, including CXCL12, IL-6, and TGF-β (15, 16). Despite these challenges, emerging immunotherapeutic strategies, including immune checkpoint blockade (ICB), neoantigen vaccines, chimeric antigen receptor (CAR) T cells, and T-cell receptor (TCR)-engineered therapies, have opened new avenues for targeting PDAC. However, monotherapies targeting ICB have shown limited efficacy due to dense stromal components and poor immune infiltration (17, 18). Therefore, combination therapies aimed at modifying the TME, restoring antigen presentation, and enhancing immune infiltration are currently being explored (19, 20). Despite significant progress in understanding PDAC biology, most existing reviews have focused on individual aspects of PDAC immunotherapy, such as ICB, the TME, adoptive cellular therapies (ACT), or cancer vaccines (21–23). In this review, we highlight recent advances in immunotherapeutic strategies for PDAC, specifically focusing on i) the molecular mechanisms fostering their immunogenicity, ii) antigen presentation defects and neoantigen targeting, iii) ICB and combination approaches, iv) predictive biomarkers of immunotherapy response, and v) promising insights from preclinical and clinical trials. A greater understanding of PDAC’s immunosuppressive mechanisms could drive the development of effective therapeutic options, improving clinical outcomes for PDAC patients.

## 2 Phenotypic, spatial, and epigenetic heterogeneity in PDAC

PDAC exhibits extensive phenotypic heterogeneity, reflected in distinct transcriptional subtypes: classical, basal-like/squamous, and hybrid/intermediate, each associated with different oncogenic drivers, therapeutic susceptibilities, and clinical outcomes. PDAC typically arises through a stepwise evolutionary process from precursor lesions, such as pancreatic intraepithelial neoplasia (PanIN) and intraductal papillary mucinous neoplasms (IPMN). While the transition from PanIN to PDAC is driven by the sequential accumulation of mutations in *KRAS*, *CDKN2A*, *TP53*, and *SMAD4*, the transformation from IPMN to PDAC is frequently associated with additional mutations in *GNAS* and *RNF43*. Recent transcriptomic analyses have defined these subtypes and revealed that they co-exist within individual tumors, with cancer cells capable of transitioning between epithelial and mesenchymal states in response to environmental cues or therapy-induced stress (24–26). In this context, the classical subtype is characterized by a well-differentiated/epithelial phenotype, chemosensitivity, and a better prognosis; the basal-like subtype exhibits a poorly differentiated/mesenchymal phenotype, chemoresistance, and a poor prognosis. However, the hybrid or intermediate subtype exhibits a mixed transcriptional state and phenotypic plasticity, transitioning between classical and basal-like phenotypes (3). This plasticity contributes to intratumoral diversity, metastatic potential, chemoresistance, and immune evasion. Simultaneously, PDAC exhibits distinct spatial heterogeneity, characterized by sectionalized tumor-stromal architecture, hypoxic gradients, and immune cell exclusion zones. The dense desmoplastic stroma, driven by CAFs, creates physical and biochemical barriers that limit T cell trafficking and promote immune evasion (10, 27). Spatially resolved transcriptomic and imaging studies have revealed that CD8^+^ T cells frequently localize to peritumoral regions or tertiary lymphoid structures. In contrast, tumor cores are enriched in immunosuppressive myeloid cells and extracellular matrix components that support the exclusion of effector T cells (28, 29). This spatial isolation of immune effector cells directly impacts antigen presentation, interferon signaling, and responsiveness to immunotherapy.

These phenotypic and spatial characteristics are underpinned by widespread epigenetic reprogramming that influences tumor cell identity and immune interactions. Abnormal DNA methylation, histone modifications (such as EZH2/PRC2 activity and HDAC-dependent silencing), long non-coding RNAs (lncRNAs), and chromatin remodeling complexes determine the transcriptional accessibility of pathways involved in epithelial-to–mesenchymal transition (EMT), cytokine secretion, MHC-I antigen presentation, and interferon responses (30–32). Epigenetic regulators such as BRD4, EZH2, and KDM6A are often mutated in PDAC, thereby affecting transcriptional subtype switching and the repression of immune genes (33–35). Importantly, epigenetic silencing of antigen-presenting machinery and chemokines leads to T cell exclusion and the “cold” immunophenotype typical of PDAC. The co-occurrence of phenotypic diversity, spatial organization, and epigenetic dysregulation creates a complex TME that contributes to PDAC’s low immunogenicity and treatment resistance to ICB.

## 3 Factors contributing to poor immunogenicity in PDAC

### 3.1 Tumor immune microenvironment

**Figure 1:**
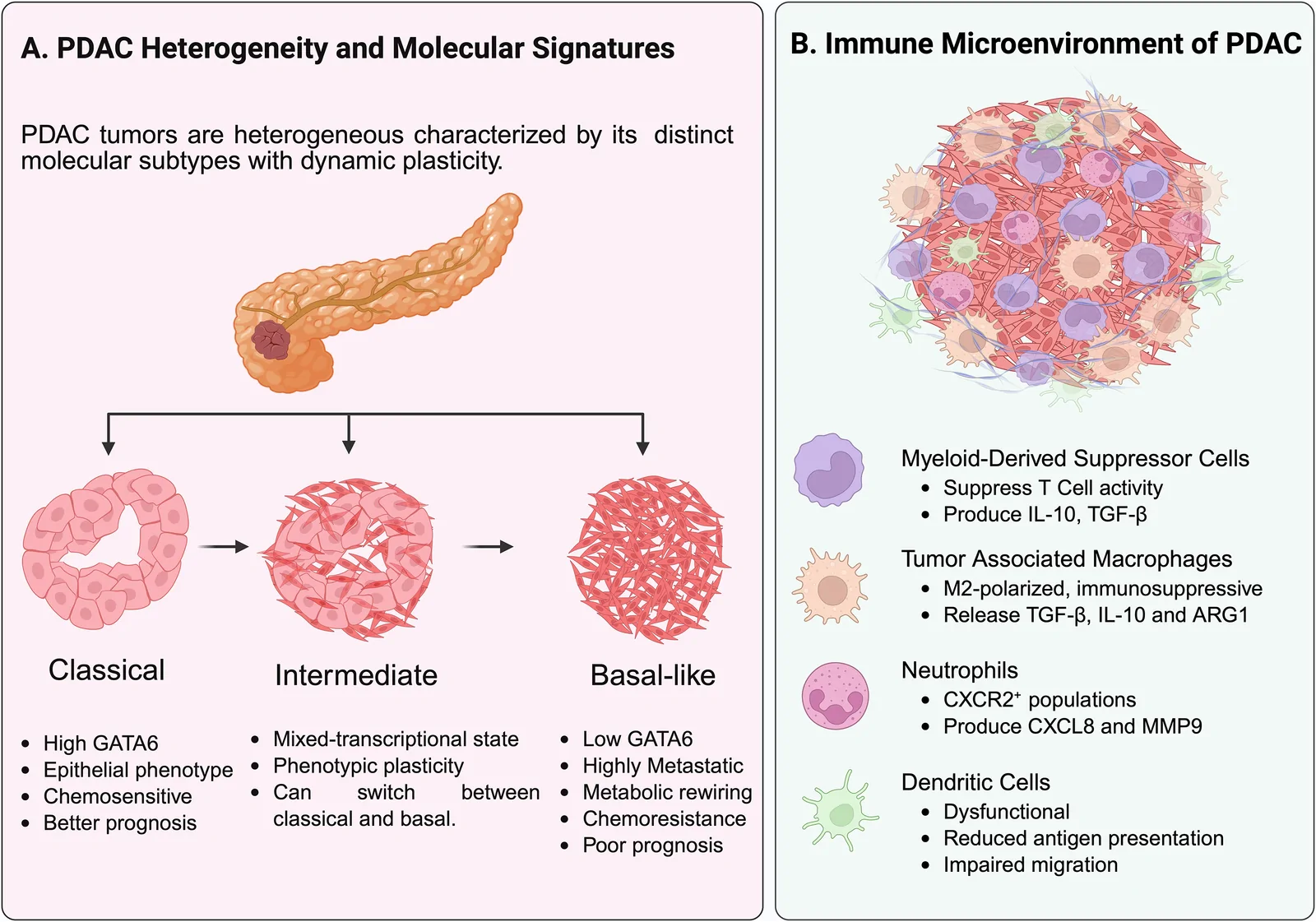
Tumor heterogeneity and immune microenvironment of PDAC. **(A)** PDAC exhibits marked molecular heterogeneity, including classical, intermediate, and basal-like subtypes with phenotypic plasticity that influences tumor behavior, treatment response, and prognosis. **(B)** The PDAC tumor microenvironment is highly immunosuppressive, enriched with myeloid-derived suppressor cells (MDSCs), tumor-associated macrophages (TAMs), neutrophils, and dysfunctional dendritic cells that collectively inhibit effective anti-tumor immunity. Together, tumor molecular signatures and plasticity shape immune composition and immune evasion in PDAC. Created using https://www.biorender.com.

PDAC exhibits significant heterogeneity in its immune landscape, comprising a wide range of innate and adaptive immune cells, many of which play tumor-promoting roles (**Figure 1**). Myeloid-derived suppressor cells (MDSCs), M2-skewed subsets of tumor-associated macrophages (TAMs), and neutrophils dominate the PDAC stroma, acting collectively to suppress cytotoxic T cell activity, thus promoting immune evasion and tumor progression (12). In addition to conventional TAM populations, recently identified TAM subsets characterized by markers such as *SPP1, TREM2, MARCO*, and *C1Q* have emerged as key regulators of PDAC progression. These macrophage subsets contribute to angiogenesis, phagocytosis, immunosuppression, and tumor promotion, and influence therapeutic responses, as demonstrated by recent single-cell and spatial transcriptomic studies (36–39). Although present, dendritic cells (DCs) are often dysfunctional and exhibit impaired antigen presentation capabilities (10). On the adaptive immune system side, cytotoxic CD8^+^ T cells and CD4^+^ helper T cells are typically either excluded from the tumor nests or, if present, exhibit an exhausted phenotype. Regulatory T cells (Tregs) further contribute to the immunosuppressive TME (40). Collectively, these immune cell populations favor immune exclusion, resistance to checkpoint blockade, and overall poor immunotherapeutic responsiveness in PDAC. In a recent single-cell multi-omics analysis, infiltrating CD45^+^ cells across PDAC patient tumors were immunophenotyped as either myeloid-enriched or adaptive-enriched. Clinically, patients with myeloid-enriched tumors were associated with poor overall survival than those with adaptive-enriched tumors, highlighting the critical prognostic role of PDAC immune composition among patients (10). An 18-gene immune signature predicted PDAC prognosis more accurately than the conventional TNM (Tumor, Nodes, and Metastasis) staging, with high-risk tumors showing M2-skewed, high-purity microenvironments (41). Macrophage-derived IL6R expression correlated with survival, and attenuated IL-6/IL6R signaling favored M2-like macrophage differentiation, highlighting IL6R as a potential immunotherapeutic target (42). Spatially, the presence of a high number of myeloid cells, such as CD15^+^ ARG^+^ granulocytes and M2-like macrophages, in close proximity to tumor cells is associated with reduced CD8^+^ T cell infiltration, leading to poor outcomes in PDAC patients (12). A single-cell transcriptomic study of the peripheral blood of a PDAC patient showed a relative reduction in the T cell population (CD45^+^CD3^+^) and an increase in myeloid lineage cells compared with healthy controls, implicating an immune shift toward a suppressive myeloid predominance during tumor development (11).

As in other cancers, myeloid cells and macrophages are recruited by chemokines and cytokines, including CCL2, CXCL1, CXCL5, CXCL8, CSF1, IL-6, and GM-CSF, secreted by PDAC cells (43–46). In PDAC, immunosuppressive tumor-associated neutrophils (TANs), such as Nectin2^+^ and OLRI^+^ TANs, have been observed to negatively correlate with CD8^+^ T cell infiltration and are associated with worse survival outcomes. These TANs enhance CCL5 expression, which recruits Tregs and promotes T cell exhaustion (via upregulated Nectin2 signaling). Inhibiting Nectin2 signaling improved cytotoxic CD8^+^ T cell function and suppressed tumor progression in Pdx-1-Cre (KPC) mice, a pancreas-specific PDAC model driven by oncogenic Kras and mutant p53 that develops spontaneous, aggressive pancreatic cancer resembling human disease, linking neutrophils to functional inhibition of cytotoxic immunity (47). Another layer of PDAC immune heterogeneity is the concept of immune coldness, in which many tumors fail to recruit functional effector T cells. PDAC outcomes improve when tumors allow robust CD8^+^ T-cell infiltration which, in turn, enhances the response to adoptive T-cell therapy. *In vivo*, TLR2 signaling on either host CD8^+^ T cells or adoptively transferred CAR T cells suppresses CAR T cell trafficking during adoptive T-cell therapy. Genetic or pharmacologic TLR2 blockade increases intratumoral T cells and slows tumor growth, suggesting that TLR2-targeted strategies can sensitize PDAC tumors to immunotherapy (48–51).

### 3.2 Stromal components and their role in immune suppression

In addition to immune cell composition, the dense and aberrant stromal population in PDAC serves as both a physical and biochemical barrier, favoring an immunosuppressive TME (14). The stroma is rich in extracellular matrix proteins (collagens, fibronectin, and hyaluronan), crosslinking enzymes (lysyl oxidase), fibrin networks, and stromal cells (CAF and PSC) (13). Collectively, these elements hinder immune cell trafficking and promote immunosuppressive signaling (52). Using a microfluidic 3D PDAC organoid/PSC co-culture model, stromal cells have been shown to protect the tumor from migrating immune cells. However, when the stroma was suppressed with halofuginone (a prolyl-tRNA synthetase inhibitor that affects fibroblasts), immune infiltration increased significantly (53). High-dimensional imaging mass cytometry revealed the spatial interplay between stromal and immune compartments on human PDAC sections. A study mapped the vascular microenvironment, immune cells, and stromal cells across 35 tumor microregions and found that stromal regions with dense fibroblasts lacked CD8^+^ T cells, indicating immune exclusion zones (54). A multimodal analysis comparing 27 primary tumors with 26 liver metastatic niches revealed significant anatomic site-specific differences in stromal and immune organization in PDAC patients. The metastatic lesions showed reduced fibroblast abundance, collagen deposition, and CD8^+^ T cell infiltration. Whereas CD163^+^ suppressive macrophages predominated in primary tumors, metastatic liver niches had a higher abundance of CD68^+^CCR2^+^ inflammatory macrophages. These observations suggest that the stromal population not only regulates immune-cell trafficking but also shapes the immunosuppressive microenvironment by interacting with infiltrating immune cells in a site-specific manner (55). Stromal fibroblasts and PSCs secrete immunomodulatory factors, including CXCL12, TGF-β, and IL-6, which limit T cell infiltration and promote immunosuppression (15). Specifically, desmoplastic stroma in PDAC acts as a major hurdle to T cell-mediated immunotherapies. Depletion of fibroblast activation protein (FAP), a marker of CAFs, using CAR T cells ultimately enhanced tumor susceptibility to tumor antigen (mesothelin)- targeted CAR T cells and anti-PD-1 therapy. This combined approach overcame stromal barriers and promoted infiltration of CD8^+^ T cells and NK cells (16). These findings highlight the therapeutic potential of dual targeting of stroma and tumor cells to improve immunotherapy efficacy in PDAC. Furthermore, neutrophils interact with the stroma, thereby worsening immunosuppression. In PDAC patients treated with or without neoadjuvant gemcitabine and nab-paclitaxel, a high infiltration of CD15^+^ neutrophils localized to the immature stromal cell niche was associated with poor clinical outcome (56). Transcriptomic and immunohistochemical profiling highlighted the prognostic role of CXCL8–CXCR2 chemokine axis, implying that patients with elevated CXCL8/CXCR2 signaling may be candidates for CXCR2-targeted strategies (56). Taken together, the stroma in PDAC functions as an active immune filter, excluding cytotoxic T lymphocytes and thereby favoring a myeloid-enriched immunosuppressive TME. Therefore, reprogramming or reshaping the stromal-immune barrier could be a key strategy to achieve immunotherapeutic success.

## 4 Therapeutic targets in PDAC immunotherapy

### 4.1 Targeting tumor-intrinsic antigens

Tumor-associated antigens (TAAs) and tumor-specific antigens (TSAs) are distinct targets on tumor cells that can be recognized by T cells and antibodies. One of the most studied TAAs is mesothelin (MSLN), which is minimally expressed in normal tissues but overexpressed in 90% of PDAC, making it a therapeutic target (57). When MSLN-targeting CAR T cells were used in patients with metastatic PDAC, disease was stabilized in 2 of 6 patients, with no effect on the primary tumor, highlighting efficacy and target engagement (58). On the other hand, TSAs are rare because they are inconsistently expressed in most tumor cells, limiting their potential as immunotherapeutic targets. A recent study identified 30% of cryptic, or non-canonical, HLA class I-bound peptides (ncHLAp) in pancreatic cancer as TSAs, which are cancer-restricted in expression and not detected in any healthy tissues. These ncHLAp demonstrated robust immunogenicity when assessed using *ex vivo* T cell priming and expansion (59). Concurrently, administration of the personalized mRNA neoantigen vaccine (Autogene Cevumeran) post-resection in PDAC patients induced specific CD8^+^ T cell responses against 20 predicted neoantigens and increased median recurrence-free survival (RFS) to up to 19 months (20). A recent phase I study evaluated a pool of synthetic long-peptide vaccines targeting 6 common mutant KRAS variants (G12V, G12A, G12R, G12C, G12D, and G13D) in combination with ipilimumab and nivolumab in patients with resected PDAC. With only grade 1–2 vaccine-related adverse events, it induced significant mutant KRAS-specific T-cell responses in 11 of 12 patients and tumor-specific T-cell responses in 10 of 12 patients in peripheral blood. Although the vaccines showed favorable safety and immunogenicity, they induced only weak immunogenicity against the KRAS G12D variant, a common mutation in 40% of PDAC cases, highlighting the need to improve the immunogenicity against this particular variant. As these findings are from a small phase I study primarily focusing on safety and immunogenicity, larger trials are necessary to assess their clinical efficacy and patients’ survival (60, 61). Similarly, a retrospective study evaluated the efficacy of personalized neoantigen peptide-pulsed DC (Neo-P DC) vaccine in patients with resected PDAC and demonstrated neoantigen-specific T cell responses in 13 of 16 patients (81.3%). Of note, seven patients in an adjuvant settings group responded better to vaccinations than nine patients in the post-operative recurrence settings group, highlighting that the timing of vaccination is crucial. Remarkably, seven responders in the postoperative recurrence settings group survived up to 36 months, whereas the OS of pancreatic cancer patients with post-operative recurrence is 14 months with standard-of-care treatment. Even though these findings are promising for personalized DC vaccines in PDAC, the timing and challenges of intranodal administration warrant prospective, large-scale clinical validation (62, 63). Therefore, this evidence suggests that PDAC offers both TAAs and TSAs as immunotherapeutic targets to stimulate anti-tumor T cell responses. However, TSAs are gaining traction because of their specificity when targeted with vaccines or ACT, which could help overcome immunosuppression in PDAC.

### 4.2 Enhancing antigen presentation

**Figure 2:**
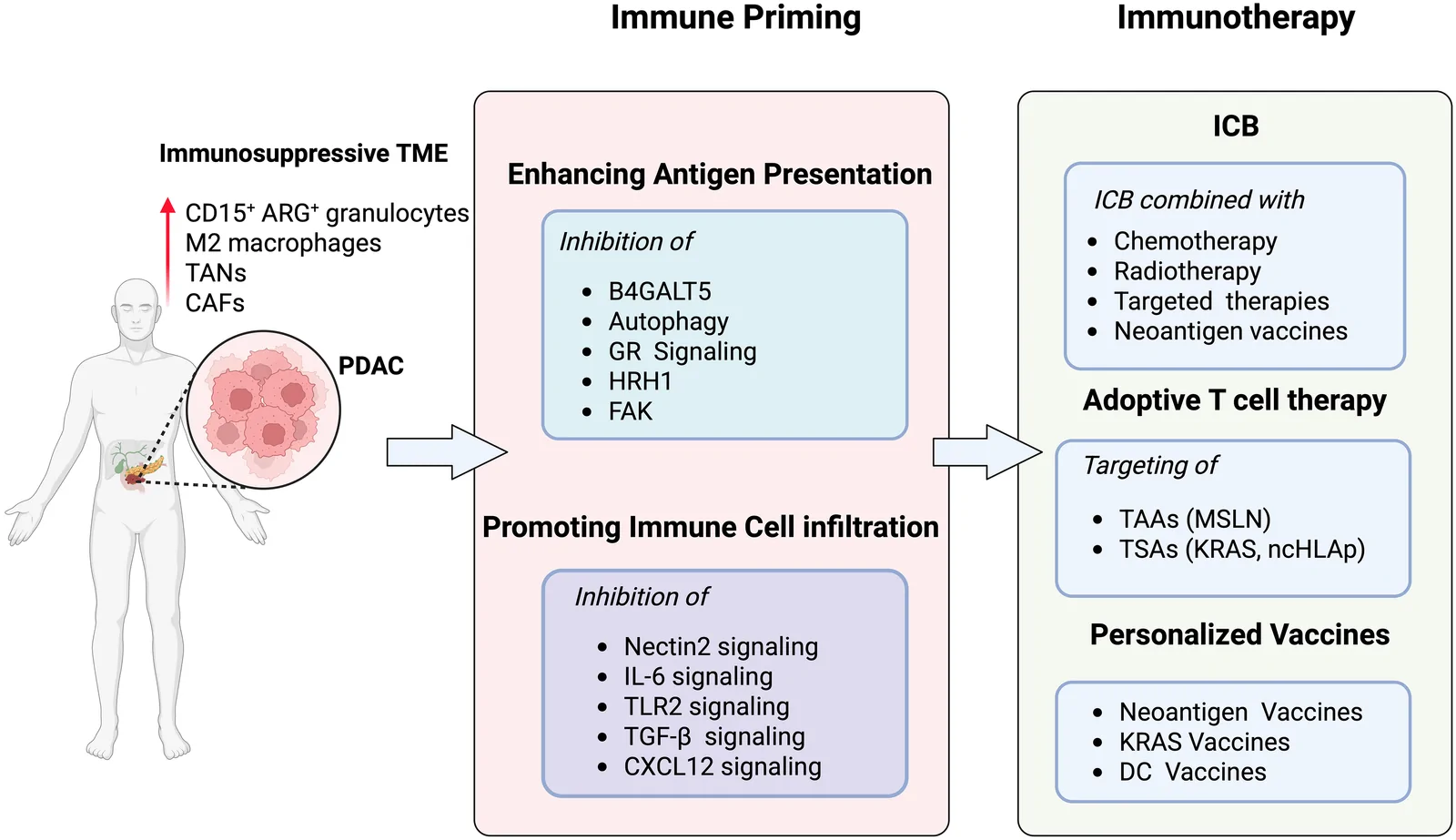
Therapeutic strategies to enhance antigen presentation, promote immune-cell infiltration, and improve immunotherapy responsiveness in pancreatic ductal adenocarcinoma. Left: PDAC is characterized by a profoundly immunosuppressive tumor microenvironment enriched with myeloid cells and cancer-associated fibroblasts (CAFs). Middle: Immune priming strategies restore antigen presentation by inhibiting B4GALT5, autophagy, glucocorticoid receptor (GR), HRH1, and focal adhesion kinase (FAK), while promoting immune-cell infiltration by targeting Nectin2, IL-6, TLR2, TGF-β, and CXCL12 signaling. Right: These approaches enhance the efficacy of immune checkpoint blockade (ICB), adoptive cellular therapies targeting tumor-associated antigens (TAAs), tumor-specific antigens (TSAs), and personalized vaccine strategies, collectively aiming to convert immunologically “cold” PDAC into an immune-responsive tumor. Created using https://www.biorender.com.

Effective targeting of TSAs requires efficient presentation of TSAs to T cells via MHC molecules, induction of co-stimulatory signaling pathways, and engagement of appropriately activated DCs. As with other cancers, PDAC tumors compromise antigen presentation by downregulating MHC class I and TAP (transporter of antigen presentation) protein expression (8, 64), thereby forming a physical barrier to immune infiltration. Within this context, focal adhesion kinase (FAK), which is highly expressed and activated in PDAC tumors, suppresses antigen presentation by transcriptionally downregulating genes involved in antigen processing. Inhibition of FAK restored tumor antigen presentation and T cell-mediated killing in PDAC models both *in vitro* and *in vivo* (65). As previously described, personalized mRNA neoantigen vaccines were designed to engage antigen-presenting cells and prime both CD4^+^ and CD8^+^ T cells in PDAC patients. Responders showed clonal expansion of neoantigen-specific long-lived CD8^+^ T cells with an estimated lifespan of 7.7 years, highlighting a durable immune memory (20, 66). In preclinical KPC mouse models, combining antigen-targeting approaches with immune modulators (CD40 agonist, PD-1 antagonist, and TLR agonist) showed enhanced DC activation and T cell priming, thereby boosting anti-tumor immunity (49, 67). Taken together, patients with intact antigen-presentation machinery (HLA-I, TAP, β2M) responded better to immunotherapy, highlighting the importance of assessing antigen-presentation capability in PDAC (**Figure 2**). It is not surprising, therefore, that the strategies deployed to improve antigen presentation in PDAC have focused on i) restoring HLA-I expression on tumor cells, ii) enhancing DC function, and iii) modulating the TME to allow the infiltration of APCs and T cells into tumor regions (**Table 1**).

**Table 1:** Mechanisms of antigen presentation dysregulation in pancreatic ductal adenocarcinoma and emerging therapeutic strategies to restore antitumor immunity.

Study model	Mechanism affecting antigen presentation	Therapeutic strategy	Clinical relevance	Key insight	Ref.
Human PDAC and mouse models	MHC-I degradation through the autophagy–lysosome pathway	Dual ICB (anti-PD-1/CTLA-4) ± autophagy inhibition	Restores antigen presentation and sensitizes tumors to immune checkpoint blockade	PDAC evades immune surveillance by selectively degrading MHC-I through autophagy	(8)
PDAC cell lines (mechanistic study)	MHC-I retention in the endoplasmic reticulum followed by autophagic sequestration	Targeting defective antigen processing	Potential strategy to restore antigen presentation and overcome ICB resistance	A multi-protein complex misroutes MHC-I to lysosomes, limiting surface antigen presentation	(84)
Human PDAC datasets and mouse models	HLA-ABC (class I) expression inversely correlated with histamine receptor HRH1	HRH1 inhibition	Enhances HLA-I expression, CD8^+^ T-cell infiltration, and ICB responsiveness	HRH1 suppresses antigen presentation and promotes immune evasion	(85)
Metastatic PDAC (case report and ongoing clinical trials)	HLA restriction required for TCR-T efficacy (HLA-C*08:02; HLA-A*11:01)	KRAS-specific TCR-engineered T-cell therapy	Patient selection depends on compatible HLA genotype for effective antigen recognition	Tumor regression occurred only when the patient’s HLA matched the engineered TCR	(86)
Human PDAC samples and mouse models	MHC-I downregulation mediated by B4GALT5 activity (β2M-free HLA-I phenotype)	B4GALT5 inhibition	Restores MHC-I expression and enhances CD8^+^ T-cell-mediated cytotoxicity	B4GALT5 suppresses antigen presentation and promotes immune escape	(87)
Human and mouse PDAC models	Glucocorticoid receptor suppresses MHC-I and β2-microglobulin while inducing PD-L1	Glucocorticoid receptor inhibition	Restores antigen presentation and improves sensitivity to immune checkpoint blockade	GR signaling simultaneously impairs antigen presentation and promotes adaptive immune resistance	(88)
Resected PDAC patients (clinical trial)	Functional dendritic cell-mediated antigen presentation	Adjuvant dendritic cell vaccine plus standard of care	Improved relapse-free survival through enhanced T-cell priming	Effective dendritic cell antigen presentation supports durable antitumor immunity	(121)

APM, antigen presentation machinery; β2M, beta-2 microglobulin; B4GALT5, beta-1, 4-galactosyltransferase 5; CD8, cluster of differentiation 8; CTLA-4, cytotoxic T-lymphocyte–associated protein 4; DC, dendritic cell; ER, endoplasmic reticulum; GR, glucocorticoid receptor; HLA, human leukocyte antigen; HLA-I, human leukocyte antigen class I; HLA-ABC, human leukocyte antigen A, B, and C; HRH1, histamine receptor H1; ICB, immune checkpoint blockade; MHC-I, major histocompatibility complex class I; NK, natural killer; PD-1, programmed cell death protein 1; PD-L1, programmed death-ligand 1; PDAC, pancreatic ductal adenocarcinoma; RFS, recurrence-free survival; SOC, standard of care; TCR, T cell receptor; TCR-T, T cell receptor–engineered T cell therapy.

### 4.3 Immune checkpoint blockade

Immune checkpoint inhibitors (ICIs) that target the PD-1/PD-L1 and CTLA-4 pathways have revolutionized treatment for various cancers by reactivating T-cell-driven antitumor responses. However, their effectiveness in PDAC has been limited, especially when used as monotherapy. Studies on pembrolizumab (anti-PD-1), ipilimumab (anti-CTLA-4), and dual PD-L1/CTLA-4 blockade generally show only modest results, with significant responses mainly seen in rare biomarker-positive groups, such as tumors with high microsatellite instability (MSI-H) or mismatch repair deficiency (dMMR) (17, 18, 68). Notably, PDAC’s highly immunosuppressive microenvironment underlies the limited efficacy of ICI. Given PDAC’s immunosuppressive TME, combination therapeutic strategies that simultaneously target immune checkpoint and tumor-intrinsic signaling would be more beneficial. Specifically, oncogenic IL-6/STAT3 and KRAS-MAPK act as central regulators in promoting tumor proliferation and survival and favor a pro-tumor microenvironment. In an orthotopic KPC murine model, dual blockade of IL-6 and CTLA-4 significantly inhibited tumor growth and progression. Mechanistically, the dual therapy induced CD4^+^ T cells to secrete IFN-γ which, in turn, increased tumor-intrinsic CXCR3 production, thereby enhancing anti-tumor immunity (19). In the clinic, dual blockade of IL-6 and PD-1 in patients with metastatic PDAC (mPDAC) elicited no response and was associated with an acceptable safety profile (69). Because the immune characteristics of the primary and metastatic tumors differ significantly (55), this likely would have contributed to the results of the above study. Although IL-6/STAT3 signaling is a rational target for combination therapies, large-scale clinical studies are necessary to determine whether the patient’s prognostic stage grouping is crucial for favorable clinical outcomes. On the other hand, a combination of MEKi (Trametinib), STAT3i (Ruxolitinib), and anti-PD-1 (Nivolumab), targeting KRAS-MAPK signaling and immune checkpoint pathways, significantly improved anti-tumor immunity and survival in the PKT mice model, an aggressive and immunosuppressive PDAC model driven by Kras activation and loss of Trp53 and Tgfbr2 (70). Based on these insights, an early-phase clinical study is evaluating the safety profile, immunogenicity, and antitumor activity (71). Although the results are not yet published, this trial will provide clinical insights into whether simultaneously targeting tumor-intrinsic oncogenic signaling and immune checkpoint pathways can remodel the PDAC tumor microenvironment and improve clinical outcomes.

## 5 Predictive biomarkers of immunotherapy response in PDAC

Despite recent advances, the clinical application of predictive biomarkers in PDAC remains limited (**Table 2**). At present, MSI-H/dMMR is the sole biomarker guiding ICI therapy, but it occurs in only 1–2% of cases. Similarly, high tumor mutational burden (TMB) is rare and relevant to a small subset of patients (18, 72–74). Evidence shows that biomarker status can change over the course of the disease, highlighting the need for serial molecular profiling in some patients. A recent case report of a patient with borderline resectable PDAC highlighted the development of an MSI-H phenotype in the tumor, which was detected via a liquid biopsy, after 20 months of chemotherapy. Based on this finding, the patient was given pembrolizumab and sustained a PFS of 13 months. Of note, MSI status varied between two commercial liquid tests, highlighting current issues with assay consistency and interpretation of new biomarkers (74). These findings underline the importance of ongoing biomarker assessment and the necessity for standardized, validated molecular testing before broad clinical use.

**Table 2:** Established and emerging predictive biomarkers of immunotherapy response in pancreatic ductal adenocarcinoma and their clinical relevance.

Biomarker category	Biomarker	Direction	Immunotherapy relevance	Clinical status	Ref.
Genomic biomarkers	MSI-H/dMMR	↑ response	High neoantigen load drives strong sensitivity to PD-1 blockade	Actionable	(72, 73, 122)
	High TMB	↑ response	Increased mutational burden enhances neoantigen-driven response to checkpoint inhibitors	Selectively actionable	(75)
	KRAS mutations	Variable	Facilitates selection of patients for KRAS-targeted vaccines and mutation-specific adoptive cellular therapies	Investigational	(60)
	Neoantigen quality/IR-neoAg load	↑ response	Improves identification of patients likely to respond to immune checkpoint blockade and enables personalized vaccines and adoptive T-cell therapies	Investigational	(123)
Immune biomarkers	PD-L1 expression	Variable	Reflects adaptive immune resistance; may enrich for response to PD-1/PD-L1 inhibitors, but has limited predictive value in PDAC	Investigational	(77, 124)
	CD8^+^ TILs	↑ response	Indicates pre-existing antitumor immunity and predicts benefit from checkpoint blockade and vaccine strategies	Investigational	(77, 79)
	IFN-γ signature	↑ response	Marks inflamed (“hot”) tumors associated with sensitivity to PD-1 blockade	Investigational	(78)
	CCL4, CCL5, CXCL9, and CXCL10	↑ response	Promote recruitment of effector T cells and enhance responsiveness to checkpoint inhibitors	Investigational	(125)
	Tertiary lymphoid structures (TLSs)	↑ response	Organized immune niches that enhance antigen presentation, T-cell priming, and adaptive antitumor immunity; associated with improved survival and increased immunotherapy responsiveness	Investigational	(83)
	HLA-I/β2-microglobulin	↑ response	Preserved antigen presentation enhances cytotoxic T-cell recognition, whereas loss contributes to immune escape and resistance to checkpoint blockade and T-cell therapies	Investigational	(8, 84–88)
	Tregs/MDSCs	↓ response	Suppress cytotoxic T-cell activity and promote resistance to immunotherapy	Investigational	(126)
	TAMs (M2 phenotype)	↓ response	Promote immune evasion and T-cell exclusion; potential therapeutic target through CSF1R inhibition	Investigational	(80, 127)
Emerging biomarkers	Microbiome	↑ response	Specific gut and intratumoral microbial communities regulate antigen presentation, T-cell activation, and responsiveness to checkpoint blockade	Investigational	(93)
	ctDNA	Longitudinal	Enables real-time monitoring of minimal residual disease, treatment response, and emergence of resistant clones during immunotherapy	Emerging	(95, 96)
	Neutrophil-to-lymphocyte ratio (NLR)	High = ↓ response	Reflects systemic immunosuppression and is associated with poorer immunotherapy outcomes	Prognostic	(128, 129)

MSI-H, microsatellite instability–high; dMMR, mismatch repair deficiency; TMB, tumor mutational burden; PD-L1, programmed death-ligand 1; TILs, tumor-infiltrating lymphocytes; IFN-γ, interferon gamma; TAMs, tumor-associated macrophages; MDSCs, myeloid-derived suppressor cells; ctDNA, circulating tumor DNA; NLR, neutrophil-to-lymphocyte ratio."↓" means decreased and "↑" means increased.

Emerging biomarkers strive to capture the presence of pre-existing anti-tumor immunity and its functional state. For instance, CD8^+^ tumor-infiltrating T lymphocytes (TILs), interferon-γ–related gene signatures, and chemokines such as CCL4, CCL5, CXCL9, and CXCL10 serve as indicators of active immune engagement and effective T-cell trafficking, and have been associated with improved responsiveness to checkpoint blockade (76–79). Conversely, biomarkers reflecting immune dysfunction or exclusion, such as elevated Tregs, MDSCs, and TAMs, are increasingly being explored as therapeutic targets in combination strategies designed to overcome resistance (80). To evaluate the relevance of immune-based biomarkers, a phase II trial of 105 mPDAC patients treated with chemoimmunotherapy revealed that distinct immune signatures correlated with treatment-specific survival benefits. Patients receiving nivolumab plus chemotherapy with a less immunosuppressive TME and higher levels of activated circulating T cells showed improved outcomes. Those receiving sotigalimab (CD40 agonist) plus chemotherapy benefited from increased intratumoral CD4^+^ T-cell presence, differentiated circulating CD4^+^ T cells, and APCs. No biomarker identified patients who benefited from the triple therapy, highlighting the complexity of immune interactions in the PDAC (81). Although these results suggest that immune profiling could guide patient selection, the analyses were exploratory and need prospective validation before routine use.

In addition to conventional immune-cell biomarkers, the compartmentalization of immune cells within the TME is an important factor in predicting immunotherapy response. The organized clusters of B cells, T cells, DCs, and specialized blood vessels, also called Tertiary lymphoid structures (TLSs), have shown promise as biomarkers in PDAC. In a study of 110 treatment-naive PDAC samples, higher TLS levels were linked to longer survival, increased intratumoral T-cell presence, and more active tumor-draining lymph nodes, indicating their role in supporting both local and systemic immune responses (82). Mechanistically, IL-33 activates group 2 innate lymphoid cells (ILC2s), promoting TLS formation. The presence of lymphogenic ILC2s and IL-33-expressing cells within TLSs correlates with a better prognosis, suggesting that inducing TLSs could improve responses to immunotherapy (83). Furthermore, the presence of antigen-presenting components, such as HLA class I and β2-microglobulin, has been identified as a potential predictor of immunotherapy success. In contrast, defects in antigen presentation can lead to immune evasion and reduce the effectiveness of checkpoint inhibitors and T-cell therapies (8, 84–88).

Recent work has highlighted the microbiome as a modulator of immunotherapy response in PDAC, adding another layer of biomarker complexity. Both gut and tumor-resident microbiota can influence systemic and local immune tone by regulating antigen presentation, T-cell priming, and cytokine production (89, 90). A key study found that long-term survivors (LTS) had significantly higher intratumoral microbial α-diversity compared to short-term survivors (STS) in PDAC. It also identified a microbial signature, including *Streptomyces*, *Pseudoxanthomonas, Saccharopolyspora*, and *Bacillus clausii*, which reliably predicted long-term survival in both the discovery and validation groups. Human-to-mouse fecal microbiota transplant (FMT) experiments demonstrated that microbiota from LTS could alter the tumor microbiome, enhance immune infiltration, and inhibit tumor growth, providing mechanistic evidence that the intratumoral microbiome influences antitumor immunity and disease progression (91). Since the human data were only observational and the mechanistic insights stem from murine models, larger and more geographically-diverse prospective studies are needed before these can serve as clinical biomarkers. Another study examined tumor samples from both LTS (n=17) and STS (n=13) resected PDAC and identified a relative abundance of the bacterium *Megasphaera* that positively correlated with patient survival. In the preclinical 4T1 model, a PD-1 low-response tumor, administration of *Megasphaera* and anti-PD-1 as a combination therapy inhibited tumor growth more than monotherapy. As this is preliminary evidence, the anti-tumor activity of the bacterium *Megasphaera* warrants further clinical validation (92). Based on these insights, FMT has gained interest as a means to improve immunotherapy outcomes. Recently, a case report of advanced pancreatic cancer patient demonstrated that combining FMT with γδ T cell therapy and PD-1 blockade led to a significant tumor clearance, ~93–94% reduction in CA19–9 levels (from 1206 U/mL to ~78 U/mL), elimination of circulating tumor cells, and sustained survival with good quality of life for approximately 10–12 months of follow-up (94). Mechanistically, this approach may enhance anti-tumor immunity through bidirectional interactions between the microbiota and γδ T cells, as well as reversal of T-cell exhaustion via PD-1 blockade. However, these results are from a single patient and require validation in a larger cohort.

Circulating tumor DNA (ctDNA), an emerging biomarker, has gained interest due to its ability to enable non-invasive and real-time monitoring of disease progression, treatment response, and the development of treatment-resistant in patients (95, 96). In 790 patients with pancreaticobiliary tumors, including 570 with advanced PDAC, the mutation concordance rate (mCR) between ctDNA and tissue testing was assessed, and the potential of ctDNA as a prognostic marker was evaluated. Among PDAC patients, 65.4% patients showed significant mCR, with shared KRAS and TP53 alterations between ctDNA and tissue testing. For serial ctDNA testing, ctDNA levels were measured every 2 months in a subset of 18 patients with mPDAC. With progressive disease in all patients, there was an increase in ctDNA levels of the TP53 and KRAS variants and the emergence of new TP53 subclones. These results suggest that ctDNA can be used to identify resistant clones and to evaluate treatment response in patients (97). Using a personalized tumor-informed ctDNA assay, a prospective study analyzed the feasibility of ctDNA to detect early recurrence risk in 43 resected PDAC patients. It predicted disease recurrence about 4.6 months before radiological relapse and was associated with shorter survival. Although this evidence suggests that ctDNA is a promising prognostic marker, the study is limited by a small sample size and variation in sample collection time points (98). Although ctDNA has limitations, including inconsistent tumor shedding, limited assay sensitivity, partial concordance with tissue profiles, and personalized testing, it has the potential to detect treatment response and recurrence risk and to identify resistant clones, thereby highlighting the need for further research (95, 96).

Recent progress in computational neoantigen prediction is broadening the scope of biomarker-guided immunotherapy for PDAC beyond traditional genomic markers. In addition to mutation-derived neoantigens, intron-retention-derived neoantigens (IR-neoAgs) are emerging as valuable predictive indicators. Analysis of the TCGA-PAAD (n=178) revealed that a high IR-neoAg load was independently associated with better overall survival. Additionally, tumors with both high IR-neoAg and elevated HLA class I expression exhibited gene expression patterns similar to those of high-PD-1-response melanoma tumors, suggesting that IR-neoAg load could help identify patients more likely to benefit from immune checkpoint inhibitors (99). In a supportive longitudinal study of 70 PDAC cases, LTS tumors (n=9) had fewer high-quality neoantigens due to immune-mediated editing than STS tumors (n=6), indicating that neoantigen quality and immunogenicity matter more than quantity for immune recognition (100). However, these methods remain experimental and require further clinical validation. In the context of predictive biomarkers, there is a need for a broader approach that integrates genomic, immune, microbiome, and longitudinal biomarker data to predict prognosis and improve clinical outcomes in PDAC patients precisely.

## 6 Clinical trials and therapeutic outcomes

### 6.1 Overview of recent clinical trials in PDAC immunotherapy

Recent developments in immunotherapy have transformed the treatment of aggressive cancers. Specifically, the use of antibodies targeting immune checkpoints, such as anti-PD-1/PD-L1 and anti-CTLA-4, has shown improved results in many cancer types. Especially when combined with chemotherapy and radiotherapy, Immunotherapy has yielded better outcomes (101). However, PD-L1 overexpression is rare in PDAC, limiting its broader applicability in PD-1/PD-L1 blockade despite its association with CD8^+^ TIL frequency and clinical prognosis (77). In the phase-II KEYNOTE-158 trial, the efficacy of Pembrolizumab was evaluated in patients with non-colorectal MSI-H or dMMR tumors, which account for <2% of PDAC cases. Of note, MSI-H/dMMR tumors are rare in PDAC, limiting the broader clinical use of immune checkpoint monotherapy. Among the PDAC subset (n=22), the overall survival (OS) was 4 months, with an objective response rate of 18.2%, indicating minimal benefits in the PDAC (18). Similarly, in another phase II study, Ipilimumab monotherapy was evaluated in 20 patients with mPDAC. It showed poor efficacy, with median OS and progression-free survival (PFS) of only 4.14 and 1.84 months, respectively (17). In a randomized trial of 65 patients with mPDAC, combination therapy with anti-PD-L1 (Durvalumab) and anti-CTLA-4 (Tremelimumab) was well tolerated. It resulted in a below-threshold objective response rate of 10%, compared with no response in patients receiving Durvalumab monotherapy (68).

In clinical trials with PDAC patients, the results of ICIs have not been very encouraging. However, when combined with chemotherapy, immunotherapy, and stromal modulators, treatment with ICIs improved disease outcome. In a retrospective study, advanced PDAC patients treated with anti-PD1 plus chemotherapy (n=28) had significantly longer median PFS (7.3 vs 5.8 months) and OS (12 vs 10.2 months) than those in the chemotherapy-alone group (n=29). However, the improvements remain modest, particularly among patients without liver metastasis and diabetes (102). A Phase Ib/II study using an anti-PD-1 agent with a gemcitabine/nab-paclitaxel regimen in 17 patients with mPDAC has shown promising results, with a median PFS of 9.1 months and a median OS of 15 months. When evaluating changes in tumor cell-free copy number instability (CNI) retrospectively, a significant reduction in CNI was associated with improved PFS and OS (103). A recent Phase 2a COMBAT trial evaluated a combination of chemotherapy with anti-PD-1 and anti-C-X-C motif chemokine receptor 4 (CXCR4). It demonstrated a median OS of 7.8 months in 22 patients with mPDAC who had `prior cancer therapies (104). Furthermore, not all combination therapies were successful. For instance, a combination of anti-PD-L1 with the Bruton’s tyrosine kinase inhibitor ibrutinib, which targets various oncogenic driver pathways, showed limited anti-tumor activity in a trial involving 122 PDAC patients with a history of prior cancer therapies (105). All these studies are limited by their small sample sizes and require validation in larger trials, with patients stratified by treatment response, presence of liver metastasis, and history of diabetes.

Alongside checkpoint blockade, personalized immunotherapies have advanced in recent years. Early trials of vaccines targeting neoantigens, mutant KRAS peptides, DCs, and ELI-002 have shown good safety profiles and have triggered antigen-specific immune responses (20, 60, 62, 106). In recent times, ACT, such as mutation-specific TCR T cells, CAR-T, CAR-NK, TILs, and autologous T-cell treatments, are also being tested in early clinical trials. There is ongoing recruitment for TP53-specific TCR T cells, B7-H3-targeted CAR-T cells, and dual-target CAR-NK therapies, indicating progress in biomarker-guided immunotherapy. However, it remains unclear whether these approaches will provide lasting clinical benefits due to tumor antigen diversity, challenges in immune cell movement through dense tissue, limited cell survival, and difficulties in manufacturing (107–111).

Several viral platforms, including adenovirus, herpes simplex virus type 1, and reovirus, have been investigated in PDAC. Over the past decade, early-phase trials have well established the feasibility of both intratumoral and intravenous administration of virotherapies and have shown that oncolytic viruses infect, replicate, and lyse cancer cells, eventually triggering robust anti-tumor immune responses. The fact that these viruses can be genetically engineered to be tumor-specific and their ability to enhance anti-tumor immunity increases the safety and efficacy of virotherapies (112). However, all the trial results were limited. To date, the most compelling evidence comes from the randomized phase IIb VIRAGE trial in which VCN-01, an oncolytic adenovirus engineered to express hyaluronidase was used. Treatment with VCN-01 in combination with gemcitabine and nab-paclitaxel in mPDAC patients improved OS (10.8 months vs 8.6 months), PFS (7.0 months vs 4.6 months), and duration of response (11.2 months vs 5.4 months) compared with chemotherapy alone, while maintaining a manageable safety profile (113). Based on these findings, an ongoing phase III VIRAGE trial is underway, although larger randomized studies are required before broader clinical application.

In unstratified PDAC patients, no immuno-monotherapy has shown significant clinical benefits. Therefore, future progress should rely on biomarker-based patient stratification and rational combination therapies targeting immune checkpoints, neoantigens, stromal barriers, and other immunosuppressive mechanisms. A summary of completed and ongoing clinical trials, organized by treatment modalities, is provided in **Table 3**.

**Table 3:** Representative completed and ongoing clinical trials evaluating immunotherapeutic strategies for pancreatic ductal adenocarcinoma.

Therapeutic modality	Phase	Trial ID	Treatment/Intervention	Study population	Key clinical outcomes	Major limitations	Ref.
Immune checkpoint inhibitors (ICIs)	II	NCT02558894	Durvalumab (anti-PD-L1) ± Tremelimumab (anti-CTLA-4)	65	Very limited efficacy; ORR 3.1% with combination therapy and 0% with durvalumab alone	Low response rates in unselected PDAC; limited clinical benefit	(68)
	I	NCT03977272	Modified FOLFIRINOX + Sintilimab (anti-PD-1)	55	Addition of PD-1 blockade did not significantly improve survival compared with standard chemotherapy	Small early-phase study; no clear survival advantage	(130)
	Ib/II	NCT02583477	Durvalumab + Nab-paclitaxel/Gemcitabine or + AZD5069	27 enrolled (23 treated)	Poor outcomes (OS <6 months; PFS <2 months) despite combination therapy	Small cohort; limited efficacy despite rational combinations	(131)
	Ib/II	NCT03611556	Oleclumab (anti-CD73) ± Durvalumab + Chemotherapy	213	Safe and well tolerated; modest improvements in OS and PFS	Clinical benefit remained limited in metastatic PDAC	(132)
Combination immunotherapy and stromal modulation	I	NCT04612530	Irreversible electroporation (IRE) + Nivolumab ± IMO-2125 (TLR9 agonist)	18	Enhanced immune activation and evidence of immunogenic priming	Very small cohort: efficacy, dosing, and treatment sequencing require further study	(133)
	I/II	NCT03767582	Nivolumab + CCR2/CCR5 antagonist (BMS-813160) ± GVAX after chemoradiotherapy	46	Safe and feasible, particularly in patients undergoing delayed surgery	Early-phase study with modest efficacy signals	(134)
	II	NCT04666740	Pembrolizumab + Olaparib maintenance	HRD-positive metastatic PDAC	Durable benefit observed in selected patients with BRCA1/2 or PALB2 mutations; translational analyses linked responses with higher TILs, ctDNA responses, and frameshift indel neoantigens	Biomarker-selected population; non-randomized phase II design; broader applicability remains uncertain	(114)
Cancer vaccines	I	NCT04853017	ELI-002 (Amph-CpG-7909 + Amph-peptides)	25	Early evidence of delayed relapse in patients with minimal residual disease	Early-phase study; long-term efficacy remains unknown	(106)
	I	NCT03592888	Mature dendritic cell (mDC3/8) vaccine	38	Safe and immunogenic; increased activated CD4+ T cells and estimated 2-year relapse-free survival of 64%	Small single-arm study without randomized comparison	(135)
	I	NCT04117087	Mutant KRAS synthetic long-peptide vaccine + Ipilimumab + Nivolumab	12	Significant KRAS-specific T-cell responses in 11/12 patients with robust KRAS-specific and cross-reactive T-cell responses with favorable safety	Phase I study primarily evaluating safety and immunogenicity; clinical efficacy remains to be established	(60)
Adoptive cellular therapies	I	NCT0354298	Mesothelin-targeted CAR-T cells ± Cyclophosphamide	15	Well tolerated; stable disease achieved in 11 patients	Anti-CAR antibodies limited CAR-T persistence and long-term efficacy	(107)
	I	NCT06158139	B7-H3-targeted CAR-T cells (iC9.CAR.B7-H3)	Recruiting	Ongoing evaluation of safety and tolerability	Recruiting; clinical efficacy not yet established	(108)
	I/II	NCT03192462	Autologous polyclonal Th1-polarized T cells targeting PRAME, SSX2, MAGEA4, Survivin, and NY-ESO-1	37 infused	Favorable safety; disease control rate 84.6% in chemotherapy-responsive patients; persistence of infused T cells up to 12 months; antigen spreading correlated with clinical benefit	Small non-randomized early-phase study; efficacy requires prospective validation	(109)
	I	NCT07627711	Dual-target CAR-NK cells (MSLN/MUC1 or CLDN18.2/MUC1)	Recruiting	Ongoing dose-escalation study evaluating safety, tolerability, RP2D, and preliminary antitumor activity	Recruiting; clinical efficacy not yet established	(110)
	I	NCT05877599	TP53 R175H-specific CRISPR-engineered TCR-T cells	Recruiting	Ongoing first-in-human study evaluating safety, maximum tolerated dose (MTD), recommended phase II dose (RP2D), and preliminary antitumor activity in TP53 R175H–positive solid tumors, including PDAC	Recruiting; clinical efficacy not yet established	(111)
Emerging immunomodulatory strategies	I	NCT03946800	Intratumoral IL-12 mRNA lipid nanoparticles (MEDI1191) + Durvalumab	Unspecified	Trial completed	Clinical outcome data not yet published	(136)
Oncolytic virotherapy	IIb	NCT05673811	VCN-01 (zabilugene almadenorepvec) + gemcitabine/nab-paclitaxel	112	Improved OS (10.8 vs 8.6 months), PFS (7.0 vs 4.6 months), and duration of response (11.2 vs 5.4 months) compared with standard chemotherapy; treatment was generally well tolerated	Open-label phase IIb trial; findings require confirmation in larger phase III studies before routine clinical adoption	(113)
	I	UMIN000010150	Intratumoral HF10 combined with gemcitabine and erlotinib	12 patients with unresectable locally advanced PDAC	HF10 demonstrated an acceptable safety profile with no dose-limiting toxicities attributable to the virus. Among evaluable patients, 3 achieved partial response and 4 had stable disease. Median PFS and OS were 6.3 and 15.5 months, respectively, and two patients were successfully downstaged to complete surgical resection.	Small, single-arm phase I study designed primarily to evaluate safety; efficacy findings require confirmation in larger randomized trials.	(137)

### 6.2 Efficacy and safety profiles of immunotherapeutic agents

Despite safety challenges and limited efficacy, immunotherapies remain an effective approach. These limitations largely stem from the distinctive characteristics of the PDAC TME, a complex mixture of immune and stromal cell populations with both anti-tumor and pro-tumor functions. Collectively, these components promote tumor progression and influence therapeutic responsiveness. ICIs, particularly when combined with chemotherapy, have shown limited clinical benefit in PDAC. A recent systematic review and meta-analysis of ICIs in PDAC management reported a significant survival advantage for ICI–chemotherapy combinations, with an 18% reduction in mortality risk (hazard ratio (HR) = 0.82; 95% confidence interval (CI): 0.78–0.87), consistent across multiple trials (7). These findings support the hypothesis that chemotherapy may enhance tumor immunogenicity and augment immune-mediated anti-tumor responses. However, this benefit was accompanied by only a minimal improvement in objective response rates (odds ratio (OR) = 1.10; 95% CI: 1.02–1.18), indicating that tumor regression remains limited in most patients (7). In contrast, combining ICIs with radiotherapy was associated with an increased risk of mortality (HR = 1.18; 95% CI: 1.04–1.34), highlighting potential adverse immunological effects, including radiation-induced lymphodepletion and dysregulated immune activation, and underscoring the need for careful treatment timing and patient selection. The substantial heterogeneity observed across trials (I² = 96%) further underscores the complexity of integrating ICIs with radiation-based approaches. PFS analyses showed a consistent improvement with ICIs, especially when combined with chemotherapy (HR = 2.25; 95% CI: 2.15–2.36); however, variability across studies indicates that therapeutic benefit is not uniform, supporting the need for biomarker-driven patient stratification (7).

Despite these limited benefits, ICIs have not yet produced substantial clinical outcomes in PDAC patients. However, recent biomarker-driven studies suggest that selected molecular subgroups may derive meaningful benefit from immunotherapy-based approaches. The phase II POLAR trial showed that maintenance therapy with anti-PD-1 (pembrolizumab) and PARP inhibitor (olaparib) offers extended clinical benefits for a subset of mPDAC patients with homologous recombination deficiency (HRD), particularly those with BRCA1/2 or PALB2 mutations, after platinum-based chemotherapy. Further translational analyses indicated associations among durable responses, higher TILs, ctDNA responses, and enrichment of frameshift indel neoantigens, supporting the shift toward targeted immunotherapy approaches in PDAC (114). Despite these encouraging findings, the POLAR trial should be interpreted with caution because it enrolled a highly selected molecular subgroup of patients and was conducted as a phase II, biomarker-driven study without randomized phase III validation. Consequently, confirmation in larger randomized studies is required before these findings can be generalized to routine clinical practice.

Major biological barriers, including a profoundly immunosuppressive TME, low tumor mutational burden, and the dominance of inhibitory immune checkpoints, continue to restrict effective anti-tumor immunity (7). Consequently, alternative immunotherapeutic strategies, such as ACT, including CAR-T cells, CAR-NK cells, TILs, TCR-engineered T cells, and cytokine-induced killer (CIK) cells, are being actively explored. Although early-phase clinical studies have demonstrated encouraging safety and feasibility, durable clinical efficacy remains limited. In addition, ACTs are associated with unique treatment-related toxicities, including cytokine release syndrome, immune effector cell-associated neurotoxicity syndrome, and on-target/off-tumor effects, particularly with engineered T-cell therapies. Furthermore, limited persistence, inefficient trafficking through the dense desmoplastic stroma, antigen heterogeneity, and manufacturing complexity continue to restrict their broader clinical application. Consequently, future efforts should focus on improving therapeutic efficacy and safety through optimized target selection, biomarker-guided patient selection, strategies that enhance immune-cell trafficking and persistence, and rational combination therapies capable of overcoming the immunosuppressive TME (115).

## 7 Future perspectives

Future research in PDAC immunotherapy should focus on rational combination strategies that simultaneously target tumor-intrinsic oncogenic signaling, stromal barriers, and immune suppression. Integrating multi-omics, single-cell, and spatial profiling with protein-protein interaction (PPI) network analysis will be critical for identifying predictive biomarkers that enable patient stratification and guide personalized treatment (116, 117). Particular emphasis should be placed on restoring antigen presentation, enhancing T-cell trafficking, and reprogramming immunosuppressive myeloid and stromal compartments. Furthermore, combining KRAS inhibitors with ICB therapies warrants further research to address the immune-cold TME typical of PDAC (118–120). Early-phase clinical trials incorporating immune modulation with chemotherapy, targeted therapies, or neoantigen-based vaccines, alongside biomarker-driven endpoints, will be essential for improving response durability and clinical outcomes. Together, these efforts may transform PDAC from an immunologically “cold” tumor into one that is responsive to immunotherapy.

## 8 Conclusion

The complex immunosuppressive TME in PDAC has made monotherapy with existing immunotherapeutic agents, including anti-PD-1 and anti-PDL-1 monoclonal antibodies, ineffective to date. Despite these challenges, recent advances in our understanding of PDAC’s immune landscape have opened new avenues for therapeutic interventions. Emerging strategies, such as neoantigen vaccines, adoptive T-cell therapies, and ICIs used in combination, have been shown to be effective at enhancing immune infiltration and overcoming therapeutic resistance. Personalized immunotherapy, guided by predictive biomarkers and the spatial immune landscape, has recently gained traction for identifying and classifying patient subsets that would benefit from immunotherapy. Further research must focus on integrating multi-omics and single-cell approaches to understand the relationship between tumor, immune, and stromal components. Developing combination therapies that can reshape the TME would be critical. Additionally, early-phase clinical trials that incorporate immune modulation, biomarkers, and existing treatments would pave the way for effective, personalized treatment options. With these findings, there are opportunities to transform PDAC treatment options to achieve more reliable and effective clinical outcomes.
